# Supplementary material for: Comparison of sequential data analysis and functional data analysis for locomotor adaptation
Source: PLoS One. 2025 Aug 29;20(8):e0329940. doi: 10.1371/journal.pone.0329940 (PMC12396696; doi:10.1371/journal.pone.0329940)
Supplement: S1 File — A full description of the dynamically weighted particle filter algorithm. (PDF) [file pone.0329940.s001.pdf]

1 Dynamically Weighted Particle Filter Model Algorithm

Let $\mathscr{M}_t = (m_{1t}^{(1)}, \ldots, m_{Gt}^{(N_t)})$ denote the N_t MCMC samples of $\boldsymbol{\theta}_t$ for time $t = 1, \ldots, T$, generated by Gibbs sampler based on the full conditional distributions described in Section **??**.

Using the DWPF for $\boldsymbol{\lambda}_{1:t}$ we assume the Markovian structure of $\boldsymbol{\lambda}_t$, that is $p(\boldsymbol{\lambda}_t|\boldsymbol{\lambda}_{1:t-1}) = p(\boldsymbol{\lambda}_t|\boldsymbol{\lambda}_{t-1})$ and $p(\boldsymbol{y}_t|\boldsymbol{y}_{1:t-1}) = p(\boldsymbol{y}_t|\boldsymbol{y}_{t-1})$. Let $q(\boldsymbol{\lambda}_t|\boldsymbol{\lambda}_{t-1}, \boldsymbol{y}_{t-1}, \boldsymbol{y}_t)$ be the proposal distribution to update $\boldsymbol{\lambda}_t$, and let $(\mathscr{L}_t, \mathscr{W}_t)$ denote the populations of MCMC samples, also called as particles, for $\boldsymbol{\lambda}_t$ and the corresponding dynamic weights, that is $\mathscr{L}_t = (\boldsymbol{\lambda}_t^{(1)}, \ldots, \boldsymbol{\lambda}_t^{(N_t)})$ and $\mathscr{W}_t = (w_t^{(1)}, \ldots, w_t^{(N_t)})$ for N_t samples at time $t = 1, \ldots, T$. Then we have the following algorithm:

Stage 1

1. (Sample) Sample $\hat{\boldsymbol{\lambda}}_1^{(i)}$ from $p(\boldsymbol{\lambda}_1|\boldsymbol{y}_1)$, and set $\hat{w}_1^{(i)} = 1$ for $i = 1, \ldots, N_0$. These form the initial population $(\hat{\mathscr{L}}_1, \hat{\mathscr{W}}_1)$ with the initial population size N_0.
2. (DWIS) Generate $(\mathscr{L}_1, \mathscr{W}_1)$ from $(\hat{\mathscr{L}}_1, \hat{\mathscr{W}}_1)$ using DWIS, with $p(\boldsymbol{\lambda}_1|\boldsymbol{y}_1)$ as the target distribution.

Stage 2

1. (Extrapolation) Generate $\hat{\boldsymbol{\lambda}}_2^{(i)}$ from $\boldsymbol{\lambda}_1^{(i)}$, with the extrapolation operator $q(\boldsymbol{\lambda}_2|\boldsymbol{\lambda}_1^{(i)}, \boldsymbol{y}_1, \boldsymbol{y}_2)$, and set

$$\hat{w}_2^{(i)} = w_1^{(i)} \frac{p(\boldsymbol{\lambda}_1^{(i)}, \hat{\boldsymbol{\lambda}}_2^{(i)}|\boldsymbol{y}_1, \boldsymbol{y}_2)}{p(\boldsymbol{\lambda}_1^{(i)}|\boldsymbol{y}_1) q(\hat{\boldsymbol{\lambda}}_2^{(i)}|\boldsymbol{\lambda}_1^{(i)}, \boldsymbol{y}_1, \boldsymbol{y}_2)}$$

   for each $i = 1, 2, \ldots, N_1$.
2. (DWIS) Generate $(\mathscr{L}_2, \mathscr{W}_2)$ from $(\hat{\mathscr{L}}_2, \hat{\mathscr{W}}_2)$ using DWIS, with $p(\boldsymbol{\lambda}_1, \boldsymbol{\lambda}_2|\boldsymbol{y}_1, \boldsymbol{y}_2)$ as the target distribution.

$\vdots$

Stage t

1. (Extrapolation) Generate $\hat{\boldsymbol{\lambda}}_t^{(i)}$ from $\boldsymbol{\lambda}_{t-1}^{(i)}$, with the extrapolation operator $q(\boldsymbol{\lambda}_t|\boldsymbol{\lambda}_{t-1}^{(i)}, \boldsymbol{y}_{t-1}, \boldsymbol{y}_t)$ and set

$$\hat{w}_t^{(i)} = w_{t-1}^{(i)} \frac{p(\boldsymbol{\lambda}_{t-1}^{(i)}, \hat{\boldsymbol{\lambda}}_t^{(i)}|\boldsymbol{y}_{t-1}, \boldsymbol{y}_t)}{p(\boldsymbol{\lambda}_{t-1}^{(i)}|\boldsymbol{y}_{t-1}) q(\hat{\boldsymbol{\lambda}}_t^{(i)}|\boldsymbol{\lambda}_{t-1}^{(i)}, \boldsymbol{y}_t)}$$

   for each $i = 1, 2, \ldots, N_{t-1}$.
2. (DWIS) Generate $(\mathscr{L}_t, \mathscr{W}_t)$ from $(\hat{\mathscr{L}}_t, \hat{\mathscr{W}}_t)$ using DWIS, with $p(\boldsymbol{\lambda}_{t-1}, \boldsymbol{\lambda}_t|\boldsymbol{y}_{t-1}, \boldsymbol{y}_t)$ as the target distribution.

Stage 1 is to initialize the population with MCMC samples simulated from the posterior distribution of $\boldsymbol{\lambda}_1$. The initial particles are obtained through the iterations of Metropolis-within-Gibbs sampler, after burn-in iterations. At each stage, the DWPF includes two steps, extrapolation and DWIS. In the extrapolation step, the samples are

extrapolated to the current stage from the preceding stage according to the transition model (Eq. **??**). In the dynamic weighting step of DWIS, only the component $\boldsymbol{\lambda}_t$ is updated through the W-type move in (1) with the dynamic ratio $r_t^{(i)} = w_t^{(i)} \frac{p(\boldsymbol{\lambda}_*^{(i)}|\boldsymbol{\lambda}_{t-1}^{(i)},\boldsymbol{y}_{t-1},\boldsymbol{y}_t)q(\boldsymbol{\lambda}_t^{(i)}|\boldsymbol{\lambda}_*^{(i)},\boldsymbol{y}_{t-1},\boldsymbol{y}_t)}{p(\boldsymbol{\lambda}_t^{(i)}|\boldsymbol{\lambda}_{t-1}^{(i)},\boldsymbol{y}_{t-1},\boldsymbol{y}_t)q(\boldsymbol{\lambda}_*^{(i)}|\boldsymbol{\lambda}_t^{(i)},\boldsymbol{y}_{t-1},\boldsymbol{y}_t)}$. We use the weight control with the weight range $W_{\text{low}} = e^{-5}$ and $W_{\text{up}} = e^{5}$ and the weight increment 2. In this step, $\boldsymbol{\lambda}_{t+1}$ replaced by the corresponding components of $\boldsymbol{\lambda}_*$. The population $(\mathscr{L}_t, \mathscr{W}_t)$ is from the DWIS because the following identity ensures that the population is correctly weighted with respect to $p(\boldsymbol{\lambda}_{t-1}, \boldsymbol{\lambda}_t|\boldsymbol{y}_{t-1}, \boldsymbol{y}_t)$

$$\frac{p(\boldsymbol{\lambda}_*|\boldsymbol{\lambda}_{t-1},\boldsymbol{y}_{t-1},\boldsymbol{y}_t)}{p(\boldsymbol{\lambda}_t|\boldsymbol{\lambda}_{t-1},\boldsymbol{y}_{t-1},\boldsymbol{y}_t)} = \frac{p(\boldsymbol{\lambda}_{t-1},\boldsymbol{\lambda}_*|\boldsymbol{y}_{t-1},\boldsymbol{y}_t)}{p(\boldsymbol{\lambda}_{t-1},\boldsymbol{\lambda}_t|\boldsymbol{y}_{t-1},\boldsymbol{y}_t)}.$$

In the population control step of DWIS, APEPCS may need to iterate for several times based on the population size constants $N_{\text{min}} < N_{\text{low}} < N_{\text{up}} < N_{\text{max}}$. We use the population control with $N_{\text{min}} = 10000$, $N_{\text{low}} = 15000$, $N_{\text{up}} = 25000$, $N_{\text{max}} = 30000$ and the initial population $N_1 = 20000$. In our experience, an allowable number of particles in the range $[N_{\text{min}}, N_{\text{max}}]$ within 10 iterations.

The whole procedure of particle filters with DWIS to generate $\boldsymbol{\lambda}_t$ and $\boldsymbol{\theta}_t$, $t = 1, \ldots, T$, can be summarized as follows: $\mathscr{T}, \theta$,

while Y_t are collected? **do**

if $t = 1$ **then** {initialize regression, DWIS, and particles}

DWIS and Particles setup

1. Assign priors for parameters and choose tuning constants.
2. Initialize the bounds for the population size and dynamic weight.
3. Generate N_1 particles $\mathscr{M}_1$ and $\mathscr{L}_1$ through a MCMC simulation.

else {proceed to the next time for $\mathscr{M}_t$ and $\mathscr{L}_t$, $t > 1$}

Generate $\mathscr{L}_t$

1. Generate $(\hat{\mathscr{L}_t}, \hat{\mathscr{W}_t})$ through the extrapolation operator.
2. Generate $(\mathscr{L}_t, \mathscr{W}_t)$ through the DWIS operator.

Generate $\mathscr{M}_t$

3. Generate $\mathscr{M}_t$ from the full conditional distribution $p(\boldsymbol{\theta}_t|\boldsymbol{\lambda}_t^{(i)}, \boldsymbol{y}_t)$, and establish $\mathscr{L}_t$ and $\mathscr{M}_t$.

end if

end while

References

1. Liang F. Dynamically Weighted Importance Sampling in Monte Carlo Computation. Journal of the American Statistical Association. 2002;97(459):807–821.
