# Supplementary material for: Comparison of sequential data analysis and functional data analysis for locomotor adaptation
Source: PLoS One. 2025 Aug 29;20(8):e0329940. doi: 10.1371/journal.pone.0329940 (PMC12396696; doi:10.1371/journal.pone.0329940)
Supplement: Attachment — Submitted filename: Response to Reviewers.pdf [file pone.0329940.s006.pdf]

Rebuttal Letter
PONE-D-24-38528

Dear Professor Gennady S. Cymbalyuk,

Please find the enclosed our revision for "Comparison of sequential data analysis and functional data analysis for locomotor adaptation" and the response letter.

We deeply appreciate your careful review and helpful concern on the paper. With regard to the suggestions raised in the review, we have substantially improved the manuscript. In this revision, we have clarified the research question and have ensured fair comparisons. We have addressed the major concerns as follows:

- *The research question is not clearly defined, and the contribution is not articulated in relation to existing literature.*

  Thank you for your comment. We have provide more detail and clarified our research question in the introduction. In page 2 and lines 30-54, we have added to the following text. "Previous investigations have leveraged standard state space models to investigate retention factors and the learning rates in motor adaptation. Using the movement kinematics collected from individual participants over time, a state space model can be used to estimate the retention and learning factors over the course of skill learning . Such analysis has providing interesting insights into how reward and punishment alter skill learning and change motor memory. Recent work has outlined the challenges associated with state space modeling in the context of skill learning. For instance, estimating retention factors is increasingly difficult with state space models, which may subject to hidden factors that underlie motor memory. Moreover, processes of learning are sources of stochasticity that are typical not taken into consideration by these models. Other factors practical factors also play a role. Missing values or the unexpected and dynamic changes of movement patterns over time may limit the robust nature of state space modeling. Taken together, this is great need to develop new approaches to modeling robust motor learning behavior. Such models could pave the way for more accurate predication of rehabilitation outcomes."

- *A critical experimental design flaw undermines the comparison between groups: the sensory group had access to both current and target knee angle feedback, giving them a significant advantage over the other groups. This invalidates direct comparisons and weakens the interpretation of feedback effects.*

  Thank you for your comment. Presentation of the current and target feedback is a common method used in rehabilitation paradigms and has been used in previous studies (Statton et al., 2016; Roemmich et al., 2016; Cherry-Allen et al., 2018). The goal of this experiment was to determine if changes in learning and memory could be induced by using different feedback mechanisms. Moreover, other experiments utilizing reinforcement feedback in the context of skill learning In essence, we are comparing a readily used method against potential new methods. We have clarified this in the text on pages 3-4, lines 117-121, which reads: "Previous work has leveraged visual error feedback of lower extremity kinematics to alter locomotor patterns and induce learning [1,23,24]. To this end, we are utilizing the methods employed by these previous studies to compare with reinforcement feedback, to determine the effectiveness each type of feedback in altering locomotor adaptation and memory, which aligns with other studies in this domain."

  Moreover, the current methods have been published elsewhere (Hill et al., 2024).

  Statton, M. A., Toliver, A., & Bastian, A. J. (2016). A dual-learning paradigm can simultaneously train multiple characteristics of walking. *Journal of neurophysiology*, *115*(5), 2692-2700.

  Roemmich, R. T., Long, A. W., & Bastian, A. J. (2016). Seeing the errors you feel enhances locomotor performance but not learning. *Current biology*, *26*(20), 2707-2716.

  Cherry-Allen, K. M., Statton, M. A., Celnik, P. A., & Bastian, A. J. (2018). A dual-learning paradigm simultaneously improves multiple features of gait post-stroke. *Neurorehabilitation and neural repair*, *32*(9), 810-820.

  Hill, C. M., Sebastião, E., Barzi, L., Wilson, M., & Wood, T. (2024). Reinforcement feedback impairs locomotor adaptation and retention. *Frontiers in Behavioral Neuroscience*, *18*, 1388495.

- *The statistical modeling is inconsistently presented and confusing. Notation is unclear, code-derived variable names are mixed with formal equations, and indices (e.g., subject ID) are used inconsistently.*

Thank you. We have reinforced the manuscript by clarifying the presentation and standardizing the notation across the manuscript.

- *Equations (especially Eq. 1 and Eq. 6) are not properly justified or explained, and there is confusion between time-series data and step-based data, calling into question the validity of the model implementation.*

  Thank you for pointing out the ambiguity of the notation here. We have made an adjustment to the notation of our models, separating the notation for the steps, which we denote as the index t and the times those steps are taken, which we denote as τ_t.

- *Important covariates (e.g., age, sex, height, weight) were not controlled for across groups, introducing potential bias.*

  Thank you for pointing this out. We may extend our model to include those important covariates in the future. However, to focus on the effects of feedback groups in our analysis, we utilized homogeneous groups of participants in age, sex, height and weight, and the preliminary analysis for the collected showed insignificant effects of those covariates. To emphasize this, we added the following comments at the end of the Data Collection Methods section. "To ensure homogeneity in the data collection, the groups were allocated in such a way as to have a similar number of males and females in each feedback group, and walked with similar treadmill speeds. Further, in a preliminary data analysis for the homogeneity of participants in age, height and weight, one-way analyses of variance revealed there was no significant differences among the three feedback groups. For EHI, the F-statistics with 2 and 3 degrees of freedom was 2.857. Subsequently, $F = 0.116$ and p-value $= 0.891$ for Age, $F = 0.082$ and p-value $= 0.921$ for Height, $F = 0.320$ and p-value $= 0.729$ for Body Weight, and $F = 0.144$ and p-value $= 0.866$ for Step Length, respectively."

- *Figures and captions are unclear or incomplete, making it difficult to interpret key results.*

  Thank you. We have repharased the captions and have added several figures directly comparing the groups with each other to help make the results clearer.

- *The task design lacks balance across experimental groups due to unequal feedback access.*

  Thank you for your comment. We have provided more rationale for the differences in feedback presentation in a previous comment, which has been added to the text on pages 3-4, lines 115-147.

- *The methods section contains redundancies, and task details are insufficiently illustrated—reviewers suggest including figures or videos to clarify the setup.*

  Thank you. We have removed redundancies have fully illustrated task detail by reorganizing the section and have added further details in Supplementary File 1. We have also added Fig. 1. to make illustrate what was done in the experiment.

- *The control group is not clearly described, and figure labeling (especially in Figs. 1, 3, 4) is poor.*

  Thank you. We have reinforced the description of the control group at page 4, lines 121-124, such that "the Control group was provided with a vertical scale with their current and target line representing the desired maximum knee flexion angle. This target line remained in the same location throughout the experiment." We also cleaned up the figure labels.

- *The manuscript contains grammatical issues and typos.*

  Thank you. We have endeavored to correct any issues in the updated manuscript.

- *The abstract and introduction are too vague and do not frame the problem or findings clearly.*

  Thank you. We have rewritten the abstract and the introduction section that are now clarified and specifically describe the goals and findings of the paper.

- *The mathematical and algorithmic descriptions require major clarification.*

  Thank you. We have made substantial changes in equations, algorithms and their interpretations for the clearer descriptions and more uniform notations across the sections of the paper.

Please also refer to the attached response to reviewers that addresses the concerns from reviewers. The revised manuscript now has been presented with much clearer way and allows more accessibility. We hope you and reviewers find the revision fully satisfactory. Please let us know if you have any questions. Your help with the editorial process of our paper is greatly appreciated.

Sincerely yours,

Torin Quinlivan, Kacy Kane, Christopher M. Hill, and Duchwan Ryu

Response to Reviewers
PONE-D-24-38528

We deeply appreciate your careful review and helpful comments on the manuscript. In what follows we describe how we have addressed the review comments to the authors:

Reviewer #1

- *The sensory groups have advantages because they can see current and target lines of peak knee flexion angle, while reward and punishment groups can only roughly estimate deviation of target without knowing whether subject needs to more or less flex knee angle.*

  Thank you for your comment. Presentation of the current and target feedback is a common method used in rehabilitation paradigms and has been used in previous studies (Statton et al., 2016; Roemmich et al., 2016; Cherry-Allen et al., 2018). The goal of this experiment was to determine if changes in learning and memory could be induced by using different feedback mechanisms. Moreover, other experiments utilizing reinforcement feedback in the context of skill learning. In essence, we are comparing a readily used method against potential new methods. We have clarified this in the text on pages 3-4, lines 117-121, which reads:

  "Previous work has leveraged visual error feedback of lower extremity kinematics to alter locomotor patterns and induce learning [1,23,24]. To this end, we are utilizing the methods employed by these previous studies to compare with reinforcement feedback, to determine the effectiveness each type of feedback in altering locomotor adaptation and memory, which aligns with other studies in this domain."

  Moreover, the current methods have been published elsewhere (Hill et al., 2024).

Statton, M. A., Toliver, A., & Bastian, A. J. (2016). A dual-learning paradigm can simultaneously train multiple characteristics of walking. *Journal of neurophysiology*, *115*(5), 2692-2700.

Roemmich, R. T., Long, A. W., & Bastian, A. J. (2016). Seeing the errors you feel enhances locomotor performance but not learning. *Current biology*, *26*(20), 2707-2716.

Cherry-Allen, K. M., Statton, M. A., Celnik, P. A., & Bastian, A. J. (2018). A dual-learning paradigm simultaneously improves multiple features of gait post-stroke. *Neurorehabilitation and neural repair*, *32*(9), 810-820.

Hill, C. M., Sebastião, E., Barzi, L., Wilson, M., & Wood, T. (2024). Reinforcement feedback impairs locomotor adaptation and retention. *Frontiers in Behavioral Neuroscience*, *18*, 1388495.

- *The state model description is questionable. Formula (1) shows 'mean knee angle' for particular time and group as sum of knee angle and perturbation for particular time and group and subject. There is no index 'I' in left part of equation, but there is one in right part of equation. One may consider that the right part of equation was summarized across all subjects, another one may suggest different operations on omitted index. As well 'knee angle' used in right part of equation denote the knee angle at time t = 0,1,...,T in walking. While early in methods there is information about 250 steps for baseline and 500 steps for adaptation. In provided code authors used 500 samples for analysis, assuming that knee angle has one value for each step, not for each time instance as in the model description.*

Thanks. We have worked to make the model description clearer and resolving those issues by cleaning up the notation. We redefined Eq. (1) to include an index i on both sides. We also make a distinction between the index of the steps, which we denote as t and the time those steps were taken, which is now denoted as τ_t. At page 5, lines 169-174, Eq. (1) has been rewritten, "Let $\mathbb{A}_j(\tau_t)$ denote the retention factor and $\mathbb{B}_j(\tau_t)$ denote the learning rate from the participant in group j at time τ_t. The standard state space model can then be described by the response model and the state model such that, for $i = 1, \ldots, n_j$ and $j = 1, \ldots, G$,

$$\begin{aligned} &\text{Response Model:} \quad & y_{ijt} &= m_{jt} + \epsilon_{ijt}, \quad t = 0, \ldots, T, \\ &\text{State Model:} \quad & y_{ij,t+1} &= \mathbb{A}_j(\tau_t) y_{ijt} + \mathbb{B}_j(\tau_t) m_{jt}, \quad t = 0, \ldots, T-1, \end{aligned} \tag{1}$$

where m_{jt} is the mean knee angle at time τ_t for the participants in group j involving perturbation ϵ_{ijt}."

- *Figures (1,3,4) are hard to see differences, maybe it would be more clear if angles of all groups were plotted on one panel with different colors, similar to figure(5).*

Thank you for the suggestion. We've added Figure 3 which overlays the means and variances for the groups over each other to make the differences clearer like you suggested. We also added a plot of the three curves overlain in Figure 5, formerly Figure 3. Figure 7, which was previously Figure 5, actually already shows the different groups from Figure 6, previously Figure 4, overlain, which we have made clearer in the text.

- *These model descriptions problems could be fixed but comparison of sensory group with other two is not correct because sensory groups have advantages mentioned earlier.*

  Thanks you for the comment. As addressed om comment # 1, presentation of the current and target feedback is a common method used in rehabilitation paradigms and has been used in previous studies (Statton et al., 2016; Roemmich et al., 2016; Cherry-Allen et al., 2018). The goal of this experiment was to determine if changes in learning and memory could be induced by using different feedback mechanisms. Moreover, other experiments utilizing reinforcement feedback in the context of skill learning. In essence, we are comparing a readily used method against potential new methods. We have clarified this in the text on pages 3-4, lines 117-121.

Reviewer #2

- *The literature review in the introduction is very brief, and both the open problem and how the authors approach it is not clearly stated.*

  Thank you for the comment. We increased the number of references in the introductions and addressed the open problem to make it clearer. At page 2, lines 20-29, we have added "Most researchers have found that punishment feedback increases the rate of visuomotor adaptation and reward enhances motor memory of task. Conversely, other tasks have noted increased learning with reward and visual feedback compared to punishment feedback. Interestingly, in the area of locomotor adaptation, studies have found mixed effects of reinforcement. For instance, one study found that both reward and punishment impaired locomotor adaptation, while another found that punishment increased memory and reward impaired adaptation. Thus, the effect of reinforcement on locomotor learning are complex, multifaceted, and in need of a consensus and more robust analysis to understand the optimal conditions for reinforcement feedback usage."

- *The experimental description is fine and understandable, but the relevance of the experiment is not stated in the intro nor anywhere else in the paper.*

  Thank you for the comment. We added some additional context to clarify the relevance of the experiment in Data Collection Methods section. Presentation of the current and target feedback is a common method used in rehabilitation paradigms and has been used in previous studies (Statton et al., 2016; Roemmich et al., 2016; Cherry-Allen et al., 2018). The goal of this experiment was to de-

termine if changes in learning and memory could be induced by using different feedback mechanisms. Moreover, other experiments utilizing reinforcement feedback in the context of skill learning. In essence, we are comparing a readily used method against potential new methods. We have clarified this in the text on pages 3-4, lines 117-121, such that "Previous work has leveraged visual error feedback of lower extremity kinematics to alter locomotor patterns and induce learning [1,23,24]. To this end, we are utilizing the methods employed by these previous studies to compare with reinforcement feedback, to determine the effectiveness each type of feedback in altering locomotor adaptation and memory, which aligns with other studies in this domain."

- *The statistical model description is obscure to say the least and notation is not consistent, bringing serious concerns about the study (see below) – e.g., equations are not interpreted, and are only given mathematically, and it seems that the R code notation is being naively translated into the mathematical description and vice-versa, raising serious confusion.*

  Thank you. We have worked to make the notation and the model description clearer and more consistent across the methods throughout the paper. More specific concerns are addressed below in those comments.

- *There is no discussion to compare the current findings with previous works in the literature, and the conclusions are not (or do not seem to be) articulated with the results displayed in the figures. The group division is arbitrary (random), and the authors do not control for some parameters that influence the findings (e.g., body height and weight, age or sex), raising serious concerns about the statistical significance of the findings.*

  Thanks. We added some additional context in the conclusion discussing why our methods in this paper are useful and more powerful than those used in previous work in the field, at page 14, lines 380-383, "Past work in the field concerning learning and retention rates have dealt with short timescales and have relied on simpler methods of analysis, namely ANOVA. These methods are limited in properly considering large datasets collected over larger timescales and can require large computational loads."

We have addressed the major concerns and reinforced the manuscript as follows:

1. *The stated parameters for the sampled population (age, sex, body height and weight) should have been done after the division into three groups (Reward, Punishment, Con-*

trol), not prior. In this way you would have been able to test the influence of these parameters on the measured difference between groups. With only the "global" average of these parameters, you cannot safely test whether height, for example, or age, influence the knee angle for a given condition. Ideally, to minimize the effects of these parameters, you'd want each of the groups (Reward, Punishment, Control) to have matching mean age, height, weight and M-F sex ratio. Did you take this into account? If you didn't, did you consider the possibility that these variables could be statistically influencing your results? Otherwise, what is your expectation about the effect these parameters have on your task, and why?

Thanks. To focus on the effects of feedback groups in our analysis, we utilized homogeneous groups of participants in age, sex, height and weight, and the preliminary analysis for the collected showed insignificant effects of those covariates. To emphasize this, we added the following comments at the end of the Data Collection Methods section. "To ensure homogeneity in the data collection, the groups were allocated in such a way as to have a similar number of males and females in each feedback group, and walked with similar treadmill speeds. Further, in a preliminary data analysis for the homogeneity of participants in age, height and weight, one-way analyses of variance revealed there was no significant differences among the three feedback groups. For EHI, the F-statistics with 2 and 3 degrees of freedom was 2.857. Subsequently, $F = 0.116$ and p-value $= 0.891$ for Age, $F = 0.082$ and p-value $= 0.921$ for Height, $F = 0.320$ and p-value $= 0.729$ for Body Weight, and $F = 0.144$ and p-value $= 0.866$ for Step Length, respectively."

2. *Eq 1: is there an implied summation over i (participants) in the m definition? Please, use an explicit notation. Also, define $\mathbb{A}$ and $\mathbb{B}$. Are these tensors? What rank? Be more explicit and consistent with your notation. Justify Eq. 1, why would the knee angle y be required to go to the mean m? Wouldn't the learning be successful if the participant achieved the required flexing angle (30°) instead of the mean m? What is $\mathbb{A}$ and $\mathbb{B}$ from the physical point of view? What is the meaning of "retention" and "learning rate"? What kind of learning is going on? How is this learning reflected in the locomotor-related brain systems? Clarify it in the manuscript. When you say "we solve the transition model" (line 139), be explicit: what is this? Inverting Eq. 1 for each time step in order to determine $\mathbb{A}$ and $\mathbb{B}$? Also clarify it in the manuscript. What do you use $\mathbb{A}$ and $\mathbb{B}$ for? Are they displayed in some figure? What are the quantities of interest, and how are they related to $\mathbb{A}$ and $\mathbb{B}$? Do you take temporal averages of $\mathbb{A}$ and $\mathbb{B}$? Although from the results, these A and B seem to be just numbers...*

The presentation of the model is very confusing, and must be thoroughly expanded and clarified (see also the next two comments).

Thank you. Learning rate (i.e. parameter $\mathbb{B}$) refers to how fast the person adapts their movement to meet the environmental demand. Retention (parameter $\mathbb{A}$) refers to the ability to carry over or remember previous performed movement. Each of these constructs tell us something different about how the brain adapts and recalls our movement. More specifically, Parameter $\mathbb{B}$ reflects the rapid adjustment of cerebellar circuits based on the large amount of sensory prediction errors present early in adaptation. Parameter $\mathbb{A}$ reflects how the movement pattern in stored in the neural connections between the motor cortex and the cerebellum. In summation, the parameters provide us with a picture of the underlying mechanisms used by the nervous system to facilitate locomotor adaptation across a short timescale. We have added these details in to the manuscript to aid in the clarity of these parameters associated with the state space model.

We have rewritten equation (1) with clarifying statements and have added more comments for interpretation. In lines 132-166, we have rephrased the statement as "At each time, we may establish a response model to characterize a group effect as the mean of knee angles of participants in the group with random noise. Whereas, in terms of the progress of the knee angles of each participant over time, the angle of the next step can be affected by a combination of the angle of the current step and the group effect shared at the current time. In other words, we may construct a state model to project the next knee angle based on the current knee angle with the retention portion and the group effect or the common behavior with the learning portion of the group. Let $\mathbb{A}_j(\tau_t)$ denote the retention factor and $\mathbb{B}_j(\tau_t)$ denote the learning rate, respectively, from the participant in group j at time τ_t. Then the standard state space model can be described by the response model and the state model such that, for $i = 1, \ldots, n_j$ and $j = 1, \ldots, G$,

$$\begin{array}{lrcll} \text{Response Model:} & y_{ijt} & = & m_{jt} + \epsilon_{ijt}, & t = 0, \ldots, T, \\ \text{State Model:} & y_{ij,t+1} & = & \mathbb{A}_j(\tau_t) y_{ijt} + \mathbb{B}_j(\tau_t) m_{jt}, & t = 0, \ldots, T-1, \end{array} \tag{2}$$

where m_{jt} is the mean knee angle at time t for the participants in group j involving perturbation ϵ_{ijt}.

In the model (2), we may utilize two approaches to estimate the mean angles m_{jt}. Using a sequential analysis the mean knee angles are time-varying parameters and a dynamic model for parameters can be estimated for each time. On the other hand,

using a functional data analysis, the mean knee angles are considered as functional values at each time. Once the mean functions are estimated, their functional values evaluated at each time can be used. Based on the estimated mean angles, we may derive the time-varying retention factors and learning rates. Two approaches in detail are described at the following subsections."

3. *Line 162: what's "IG"? Wasn't G the number of groups (i.e., $G = 3$)? G also appears in between lines 280-281. The authors should be more specific when describing their model. If one want the main manuscript to be more concise, then a supplementary file containing a detailed specific description should be provided. Are A_{sj} and B_{sj} (line 162-163) elements of the A and B tensors? But shouldn't A be rank 3 and B rank 2? The authors also write A_{mjt}, B_{mjt} (lines 165-166). Are these "mjt" or "sj" the element indices? So are these tensors rank 2 or 3? The model description is very obscure and sloppy and not precise at all, again lacking consistency in the notation within the paper. I noticed that the authors developed their own code in R. However, if the regression method is THE MAIN novelty of this paper, this must be clearly stated in the abstract and introduction. Otherwise, if the regression for each model was simply an implementation of a previously published regression method (which seems to be the case), then there's no need to explicitly state the algorithm (e.g., lines 226-234; or eqs between lines 280-281), provided that a clear mention and reference to the original work is given. I advise the algorithm to be moved to a Supplementary File 1, and focus Section 3 in a precise and rigorous description of the models (something that is not done yet). Mathematical details must be thoroughly improved, clearly stating what each symbol means, and what is the role of subscripts (elements in tensors or just variable names? From code inspection, they look like just variable names, so everything is very confusing).*

   Thanks. "IG" is the Inverse Gamma distribution. That line was simply saying that the random variable σ_{j1}^2 follows an Inverse Gamma distribution with two shape parameters A_{sj} and B_{sj}. We have updated the notation to make it clearer throughout the manuscript.

   The algorithm description has been moved to a Supplementary File 1, and the model description has been expanded to focus more on the usage of the particle filter. We also added a citation at page 6, lines 218-219 "full description of the dynamically weighted particle 218 filter algorithm can be found in Supplementary File 1 as in [27]. 219."

4. *If these tensors have independent elements "A_{mjt}" or "B_{st}", then how can they be*

equated to a function of time with a single index for group j (eq 6)? I strongly advise a full revision of the model sections (sec 3.1 and 3.2), including a preamble in Section 3 where the authors write an Eq. 1 that generalizes both approaches (SDA and FDA), and then in each subsection, the description is then reduced to that particular case. Again, stating the details of the algorithm that was used to fit the A and B tensors is not important if it was done using a software package, and can be skipped for clarity along with citing the appropriate package. DO NOT MIX code notation with the mathematical description of the code for the sake of reproducibility and clarity. The details of the algorithm must only be stated (separately in a Supporting file) if this was a major development of you paper (which I assume it wasn't, otherwise it isn't clear and it must be highlighted in the introduction that your contribution is a new algorithm for fitting statistical models).

Thank you. The algorithm description has been moved to a Supplementary File 1, and the model description has been expanded to focus more on the usage of the particle filter. We also added a citation [27].

5. *Results: what is the "Control" group? It was not described in Methods. Is the "Sensory" the "Control" group? This must be clarified. Fig 2 has unreadable labels. Figures and Tables' captions are not informative nor descriptive enough. Lines 337-338, the authors say they use a simpler model to evaluate the performance of particle filter. This MUST BE clearly described in Methods (Section 3).*

   Thank you for the comment. We have edited the manuscript to standardized the language to refer to the "Control" group. Moreover, we have added a new figure to provide further detail about the feedback protocol.

6. *Conclusion: "Both approaches have led to compatible results, while the method based on the functional data analysis has allowed more flexible inference.". This is not clear in the manuscript. The concluding remarks are too shallow: how do your results compare to previously found results in the literature? Did you just confirm previous findings? What is your contribution? What advantages/setbacks, compared to previous works, came from your statistical methods? I do not see how the figures you showed can help answering these questions, so include a Discussion articulating the relation between the figures and your findings.*

   Thank you for the comment. We have added additional context and references of other work in the field, and clarified how our methods improve upon them. "Past work in the field concerning learning and retention rates [9, 13, 15] have dealt with short timescales

and have relied on simpler methods of analysis, namely ANOVA. These methods are limited in properly considering large datasets collected over larger timescales and can require large computational loads."

We have also addressed the other concerns:

7. *A figure or video illustrating the task and the different conditions (Reward, Punishment, Sensory) is highly advisable (even though it is written in lines 90-99). For example, what was the visual feedback? What are the walking patterns (authors state there are five distinct patterns, line 74). How could the participant know they are flexing their knees by an extra 30° (line 86)? What were the task direction read to the participants? These directions can be attached to the submission, and a Figure can be added to the main manuscript, and a video could be appended as well.*

   Thank you for your comment. The data analyzed in this study was a part of a wider study contain five walking conditions. However, we limited our analysis to the first two task conditions (Baseline, Adaptation) for this manuscript. The participants did not explicitly know to what degree they needed to increase their knee flexion to. Instead they were instructed they would have to increase it in order to meet their task goal. We have added a new task figure depicting the visual feedback seen by the participants and the task directions to the supplementary material. We have also added further details about the directions in the methods section of the manuscript.

8. *The abstract is not clear. It is too concise and does not frame the work into the big picture (e.g., what is the problem being addressed, or why is it important). It does not clearly state what the authors did to address the problem either. It only states briefly that two models were compared, but what did the authors find? What is the motivation for both of these models in relation to the open problem?*

   Thank you. We have extended the abstract to clarify the goal of paper, "Learning rates for skills such as walking may depend on circumstances or time, while incentivization with punishments or rewards may affect human skill learning. We consider a state space model for dynamically changed learning rates and figure out the effect of incentivization on the learning rates by utilizing a dynamically weighted particle filter. However, estimations of model parameters, including the learning rate, require a demanding computational burden, especially when the data are collected over a long period. To overcome computational difficulty, we utilize an efficient sequential Monte Carlo method, dynamically weighted particle filter, in the estimations of model parameters. Alternatively, we consider a functional data analysis for the learning rates and

the effect of the incentivization. Two approaches have led to reasonable estimations of learning rates. We present the estimated learning rates and the effect of incentivization on the learning rates from two approaches, as well as the comparisons of their results."

9. *Intro, lines 23-24: "The effect of reinforcement on skill learning are complex, multifaceted, and in need of a consensus." It is not clear in the introduction why it is in need of "consensus". Please, clarify it in the manuscript.*

   Thanks for the comment. We have clarified what we mean by "consensus" in the manuscript, at page 2, lines 27-29, "Thus, the effect of reinforcement on locomotor learning are complex, multifaceted, and in need of a consensus and more robust analysis to understand the optimal conditions for reinforcement feedback usage."

10. *There are many typos (e.g., line 9: "there are two TYPE of errors"; line 20: "affects" should be "effects"; line 29: "providing" − > "provided"; etc). Revise the whole text for similar problems.*

    Thanks. We have corrected as many typographical errors as we can, as well as the ones pointed out here.

11. *The authors mention "motor adaptation paradigm" (line 34 Intro), but do not detail the task. Give a quick outline of the task in the introduction before talking about "knee angles" (line 37).*

    Thanks. We have clarified the locomotor adaptation paradigm in the text. "In this paper, we consider two approaches to overcome these challenges and to more accurately estimate the retention factors and learning rate of participants during a locomotor adaptation paradigm, where participants learn a new knee flexion pattern with visual feedback of their angular kinematics, and, moreover, examine the effect of reinforcement (i.e. reward and punishment) on these rates."

12. *"The derivative of fitted curves can be used for the estimation of the retention rates". Why? Either cite a work that's done that, or quickly explain it if it hasn't been done before.*

    Thanks. We have added the reasoning to use the estimated derivative function at page 8, lines 280-285, "From the state space model (1), the group mean function m_{jt} is estimated by $\widehat{f}_j(\tau_t)$ in FDA. Regarding the state model as a regression model for the knee angles at time $t+1$ on the knee angles at time t with disturbance term $\mathbb{B}_j(\tau_t)m_{jt}$, the retention faction $\mathbb{A}_j(\tau_t)$ is a slope that represents the change rate of knee angles

from current time to the next. Hence, for the estimated mean functions, the retention factor $\mathbb{A}_j(\tau_t)$ or the change of responses by the time is estimated by $\widehat{f}'_j(\tau_t)$ in FDA."

13. *Sensor positioning and data acquisition (lines 112-114) was already stated previously (lines 81-84).*

    Thanks. We have corrected that.

14. *"only the participant IDs distinguished the entries". This is not clear: was the actual "ID" (official document of the participant) used? (probably not). So rephrase... "a number was assigned to each participant" etc.*

    Thank you for the suggestion. We made the anonymization of the participants clearer in the text: "For our work in this article, we received the previously collected data, which had already been anonymized, and ran our analyzes as a secondary data analysis. We first received the data from Dr. Christopher M. Hill, School of Kinesiology, Louisiana State University, on January 11, 2023. We had no access to any of information that could have been used to identify participants while running our analyses, the data was fully anonymized when we had received it, only anonymous numerical IDs assigned to the participants distinguished the entries."

15. *Line 148: "model in (1)". Unclear: Eq. (1) or Ref (1)?*

    Thanks. We have updated the citation style. All citations are in square brackets. Now, equations are denoted by (1) and references is denoted by [1].

16. *Fig. 1, bottom row (Punishment) has mean and variance panels switched.*

    Thanks. We have corrected it.

We thank you for the review. Your comments and suggestions were greatly helpful to improve the manuscript.
