# Supplementary material for: Comparison of sequential data analysis and functional data analysis for locomotor adaptation
Source: PLoS One. 2025 Aug 29;20(8):e0329940. doi: 10.1371/journal.pone.0329940 (PMC12396696; doi:10.1371/journal.pone.0329940)
Supplement: Attachment — Submitted filename: Response_to_Reviewers_auresp_2.pdf [file pone.0329940.s007.pdf]

Rebuttal Letter
PONE-D-24-38528

Dear Professor Gennady S. Cymbalyuk,

Please find the enclosed our revision for "Comparison of sequential data analysis and functional data analysis for locomotor adaptation" and the response letter.

We deeply appreciate your careful review and helpful concern on the paper. With regard to the suggestions raised in the review, we have addressed the concerns as follows:

- *pp.170 Since retention factor A and learning rate B do not have index (i), participants (plural) should be used instead of participant.*

  Thank you for catching that. The manuscript has been updated to "Let $\mathbb{A}_j(\tau_t)$ denote the retention factor and $\mathbb{B}_j(\tau_t)$ denote the learning rate from the participants in group j at time τ_t. ".

- *pp.173 It should be 'mean knee angle across participants' instead of 'for the participants'.*

  Thank you for catching that. The manuscript has been updated to "where m_{jt} is the mean knee angle at time τ_t across participants in group j involving perturbation ϵ_{ijt}.".

Please also refer to the attached response to reviewers that addresses the concerns from reviewers. The revised manuscript now has been presented with much clearer way and allows more accessibility. We hope you and reviewers find the revision fully satisfactory. Please let us know if you have any questions. Your help with the editorial process of our paper is greatly appreciated.

Sincerely yours,

Torin Quinlivan, Kacy Kane, Christopher M. Hill, and Duchwan Ryu

Response to Reviewers
PONE-D-24-38528

We deeply appreciate your careful review and helpful comments on the manuscript. In what follows we describe how we have addressed the review comments to the authors:

Reviewer #1

- *pp.170 Since retention factor A and learning rate B do not have index (i), participants (plural) should be used instead of participant.*

  Thank you for catching that. The manuscript has been updated to "Let $\mathbb{A}_j(\tau_t)$ denote the retention factor and $\mathbb{B}_j(\tau_t)$ denote the learning rate from the participants in group j at time τ_t. ".

- *pp.173 It should be 'mean knee angle across participants' instead of 'for the participants'.*

  Thank you for catching that. The manuscript has been updated to "where m_{jt} is the mean knee angle at time τ_t across participants in group j involving perturbation ϵ_{ijt}.".

We thank you for the review. Your comments and suggestions were greatly helpful to improve the manuscript.
